# Supplementary material for: Perceptions of women and their partners on postabortion intrauterine contraception: A qualitative study in central Uganda
Source: PLoS One. 2025 Jun 25;20(6):e0316982. doi: 10.1371/journal.pone.0316982 (PMC12192054; doi:10.1371/journal.pone.0316982)
Supplement: Attachment — Submitted filename: Reviewers Comment PONE.docx [file pone.0316982.s001.docx]

### Reviewer’s comment on: Perceptions of Women and their Partners on Postabortion Intrauterine Contraception: A Qualitative study in central Uganda

Dear editor,

I am pleased to review this manuscript for PLOS ONE. I read with great interest. I am happy to confirm this manuscript should be accepted for publication following minor revisions as highlighted in the manuscript.

I have thoroughly evaluated the manuscript.

Strengths

1) The topic is relevant which enhances the importance of the study
2) Clear objective and methodological rigor. The methodology outlines the participants recruitment, detail data collection process and instrument used according to qualitative research method. This helps establish the validity of findings.

The authors can work on the few comments highlighted with tracked changes on the manuscript.

Dr Zainab Abdulkadir (MBBS, MSc.PH, FMCFM)

.
