# Supplementary material for: Perceptions of women and their partners on postabortion intrauterine contraception: A qualitative study in central Uganda
Source: PLoS One. 2025 Jun 25;20(6):e0316982. doi: 10.1371/journal.pone.0316982 (PMC12192054; doi:10.1371/journal.pone.0316982)
Supplement: Attachment — Submitted filename: Response to reviewer comments_couple perceptions.docx [file pone.0316982.s003.docx]

P.O. Box 7072, Kampala, Uganda

Phone: +256-414-534361

e-mail: [obsgyn@chs.mak.ac.ug](mailto:obsgyn@chs.mak.ac.ug)

## COLLEGE OF HEALTH SCIENCES

## SCHOOL OF MEDICINE

## DEPARTMENT OF OBSTETRICS AND GYNAECOLOGY

16^th^ April 2025

RESPONSE TO REVIEWS COMMENTS:

***Object:* PONE-D-24-58782 *“Perceptions of Women and their Partners on Postabortion Intrauterine Contraception: A Qualitative study in central Uganda”***

With great pleasure, I’m thankful for your comments towards our manuscript. In response to the reviewers’ comments sent to us on 11^th^ April 2025, we have revised the manuscript accordingly.

| **Comment** | **Response to Comment** | **Page No. and Line** |
| --- | --- | --- |
| **Journal Requirements:**  1. When submitting your revision, we need you to address these additional requirements.    Please ensure that your manuscript meets PLOS ONE's style requirements, including those for file naming. The PLOS ONE style templates can be found at  <https://journals.plos.org/plosone/s/file?id=wjVg/PLOSOne_formatting_sample_main_body.pdf> and  <https://journals.plos.org/plosone/s/file?id=ba62/PLOSOne_formatting_sample_title_authors_affiliations.pdf>    2. Thank you for stating in your Funding Statement:  This project was supported by funds from the Swedish Research Council, (Grant 2019-04256) in partnership with Makerere University and the MakRif Project. The content is solely the responsibility of the authors and does not necessarily represent the official views of The Swedish Research Council, Makerere University or the MakRif Project. The funders provided support in the form of research expenses, but did not have any additional role in the study design, data collection and analysis, decision to publish, or preparation of the manuscript.    Please provide an amended statement that declares *all* the funding or sources of support (whether external or internal to your organization) received during this study, as detailed online in our guide for authors at <http://journals.plos.org/plosone/s/submit-now>.  Please also include the statement “There was no additional external funding received for this study.” in your updated Funding Statement.  Please include your amended Funding Statement within your cover letter. We will change the online submission form on your behalf.    3. Thank you for stating the following in the Acknowledgments Section of your manuscript:  The lead author along with the co-authors extend their gratitude to the participants, doctoral committee and the research team that enable this study to become a reality. This study was undertaken as part of the lead author’s doctoral dissertation funded by the collaboration between The Swedish Research Council and Makerere University in partnership with MakRif project.    We note that you have provided funding information that is not currently declared in your Funding Statement. However, funding information should not appear in the Acknowledgments section or other areas of your manuscript. We will only publish funding information present in the Funding Statement section of the online submission form.  Please remove any funding-related text from the manuscript and let us know how you would like to update your Funding Statement. Currently, your Funding Statement reads as follows:  This project was supported by funds from the Swedish Research Council, (Grant 2019-04256) in partnership with Makerere University and the MakRif Project. The content is solely the responsibility of the authors and does not necessarily represent the official views of The Swedish Research Council, Makerere University or the MakRif Project. The funders provided support in the form of research expenses, but did not have any additional role in the study design, data collection and analysis, decision to publish, or preparation of the manuscript.    Please include your amended statements within your cover letter; we will change the online submission form on your behalf.    4. In the online submission form, you indicated that “The dataset used and analyzed in this study are also available with the corresponding author on request.”  All PLOS journals now require all data underlying the findings described in their manuscript to be freely available to other researchers, either a. In a public repository, b. Within the manuscript itself, or c. Uploaded as supplementary information.  This policy applies to all data except where public deposition would breach compliance with the protocol approved by your research ethics board. If your data cannot be made publicly available for ethical or legal reasons (e.g., public availability would compromise patient privacy), please explain your reasons on resubmission and your exemption request will be escalated for approval.  5. Please review your reference list to ensure that it is complete and correct. If you have cited papers that have been retracted, please include the rationale for doing so in the manuscript text, or remove these references and replace them with relevant current references. Any changes to the reference list should be mentioned in the rebuttal letter that accompanies your revised manuscript. If you need to cite a retracted article, indicate the article’s retracted status in the References list and also include a citation and full reference for the retraction notice.  **Additional Editor Comments:**  Thank you for submitting this study which addresses an important knowledge gap. Overall the study is well conducted and analyzed. There are the following minor revisions that may be considered.  1. While the overall manuscript is well written there are typos and some logical flow errors. Example dare instead of dire on line 54, additional word "yet" on line 70 etc. Please run word editor to ensure better readability  2. Interviews were conducted with only English speakers. This may have introduced bias towards higher educated or better socio-economic couples. Please address this in limitations including how this may impact the interpretation of your results  3. Since both husband and wife were interviewed, it would be interesting to see how their perceptions were similar and different and on what issues  **Reviewer 1:** I commend the authors’ efforts in trying to fill an important gap in research and share such important findings. After reviewing the manuscript, here are some important points that the authors should consider: • This sentence needs revision as unintended pregnancies are not necessarily unwanted pregnancies. They are unplanned. This means the pregnancies were not intended at the time of conception, regardless of whether they were unwanted or mistimed. This argument has evolved over time. Use updated articles. • The argument on not well-documented reasons should be reviewed - “Despite proven effectiveness and safety of intrauterine contraception yet with a failure rate of less than one percent [14], uptake among Ugandan women has stagnated below two percent among married and 15% among sexually active unmarried women [15] for reasons that may not be well documented”. A quick Google search reflects otherwise (<https://bmcwomenshealth.biomedcentral.com/articles/10.1186/s12905-022-01856-1>; <https://pmc.ncbi.nlm.nih.gov/articles/PMC10022157/>)  • The paragraphs after lines 72 and 73 are confusing. The previous argument was that there may not be well documented reasons for the stagnation in the uptake of intrauterine conception among Ugandan women. However, subsequent arguments in the following paragraphs stated otherwise. I think these paragraphs should be revised to reflect exactly what the authors are trying to present in the introduction.  • I noticed that many of the references cited in the manuscript are somewhat dated. While these foundational works are undoubtedly valuable, it is also essential to incorporate more recent research to provide a comprehensive and current perspective on the topic. I recommend updating the references with more recent studies that have built upon or expanded the concepts discussed in the older articles.  • “Using purposive sampling with maximal variation, fifteen women and their partners aged between 18 and 49 years were invited to participate in the study at five public facilities in central Uganda.” What does maximal variation mean? I appreciate the descriptive details of the facilities, but I do not see the relevance in the study. This may be useful for other reports on the larger RCT study. Could you share the inclusion criteria to further highlight the level of variation?  • It was stated that the sample size was influenced by assumed saturation. Could this be clarified? Could the authors elaborate on what influenced the sample size?  • Despite the subtitle, participant recruitment details were not adequately presented in the manuscript. How were participants recruited? For the few details that were included, there was no reference to whether participants had previously consented to being contacted before contact details were retrieved from the database.  • “Fifteen in-depth interviews were conducted in English or Luganda by two interviewers and two note takers with prior experience in qualitative inquiry”. I appreciate the use of either English or Luganda to enhance quality data. Could you share why these options were presented? How did the subsequent translation of documents from one language to another impact data and its analysis? How did you mitigate the loss of the meaning of ideas during translation?  • I’m curious to know the influence of the socio-demographic details such as age and education on participants’ decisions.  • I appreciate that the analysis and the codebook development were adequately presented - showing how the codebook (subthemes and themes) was developed gives credence to this qualitative work.  • The verbatim quotes under cultural beliefs on IUDs do not necessarily support the arguments in that theme. Some of the quotes are more like responses to barriers that were presented to them, rather than the barriers that were shared by the participants.  • Review statement – “We used a semi-structured interview guide to collect information on participants’ sociodemographic characteristics, and the various barriers and motivators in the utilization of the IUDs”. I suggest overall proofreading and manuscript editing.  • A more detailed conclusion should be considered.  • Overall, the authors have presented important findings that will contribute to ongoing arguments on postabortion intrauterine contraception in Uganda and the global context if the manuscript is reviewed adequately before publication.  [Note: HTML markup is below. Please do not edit.]  Reviewers' comments:  Reviewer's Responses to Questions  **Comments to the Author**  1. Is the manuscript technically sound, and do the data support the conclusions?  The manuscript must describe a technically sound piece of scientific research with data that supports the conclusions. Experiments must have been conducted rigorously, with appropriate controls, replication, and sample sizes. The conclusions must be drawn appropriately based on the data presented.  Reviewer #1: Yes  Reviewer #2: Yes  2. Has the statistical analysis been performed appropriately and rigorously?  Reviewer #1: N/A  Reviewer #2: Yes  3. Have the authors made all data underlying the findings in their manuscript fully available?  The [PLOS Data policy](http://track.editorialmanager.com/CL0/http:%2F%2Fwww.plosone.org%2Fstatic%2Fpolicies.action%23sharing/1/010f019621df6bd3-9a05a3e1-3cd7-40b6-9590-fbe151f9217f-000000/YHU5dHbrgZa2WqVqid5VB__BRQn2tYcUrYyQszQJwx0=206) requires authors to make all data underlying the findings described in their manuscript fully available without restriction, with rare exception (please refer to the Data Availability Statement in the manuscript PDF file). The data should be provided as part of the manuscript or its supporting information, or deposited to a public repository. For example, in addition to summary statistics, the data points behind means, medians and variance measures should be available. If there are restrictions on publicly sharing data—e.g. participant privacy or use of data from a third party—those must be specified.  Reviewer #1: Yes  Reviewer #2: Yes  4. Is the manuscript presented in an intelligible fashion and written in standard English?  PLOS ONE does not copyedit accepted manuscripts, so the language in submitted articles must be clear, correct, and unambiguous. Any typographical or grammatical errors should be corrected at revision, so please note any specific errors here.  Reviewer #1: Yes  Reviewer #2: Yes  5. Review Comments to the Author  Please use the space provided to explain your answers to the questions above. You may also include additional comments for the author, including concerns about dual publication, research ethics, or publication ethics. (Please upload your review as an attachment if it exceeds 20,000 characters)  **Reviewer #2:** The manuscript is well written and the research is conducted accordingly with methodological rigor. There is no research or evidence of publication ethical issues in the this manuscript to the best of my understanding.  6. PLOS authors have the option to publish the peer review history of their article ([what does this mean?](https://track.editorialmanager.com/CL0/https:%2F%2Fjournals.plos.org%2Fplosone%2Fs%2Feditorial-and-peer-review-process%23loc-peer-review-history/1/010f019621df6bd3-9a05a3e1-3cd7-40b6-9590-fbe151f9217f-000000/YpilxRmDsrue6ATmzu7xyolNw1TkhJh2HaLLSE4xOQk=206)). If published, this will include your full peer review and any attached files.   If you choose “no”, your identity will remain anonymous but your review may still be made public.   **Do you want your identity to be public for this peer review?** For information about this choice, including consent withdrawal, please see our [Privacy Policy](https://track.editorialmanager.com/CL0/https:%2F%2Fwww.plos.org%2Fprivacy-policy/1/010f019621df6bd3-9a05a3e1-3cd7-40b6-9590-fbe151f9217f-000000/_KxiBLJVVxDA5Q9ZUVYgY0Bdm90J2bw7jObQmKz4mvA=206).  Reviewer #1: No  Reviewer #2: No  [NOTE: If reviewer comments were submitted as an attachment file, they will be attached to this email and accessible via the submission site. Please log into your account, locate the manuscript record, and check for the action link "View Attachments". If this link does not appear, there are no attachment files.]  While revising your submission, please upload your figure files to the Preflight Analysis and Conversion Engine (PACE) digital diagnostic tool, [https://pacev2.apexcovantage.com/](https://track.editorialmanager.com/CL0/https:%2F%2Fpacev2.apexcovantage.com%2F/1/010f019621df6bd3-9a05a3e1-3cd7-40b6-9590-fbe151f9217f-000000/wratWyzcq3FMPpGs9ayU7thgrCIEDUOqlk_gHfjqJcY=206). PACE helps ensure that figures meet PLOS requirements. To use PACE, you must first register as a user. Registration is free. Then, login and navigate to the UPLOAD tab, where you will find detailed instructions on how to use the tool. If you encounter any issues or have any questions when using PACE, please email PLOS at [figures@plos.org](mailto:figures@plos.org). Please note that Supporting Information files do not need this step. | Thanks so much for the feedback. The manuscript has been revised in accordance to the PLOSONE style templates as recommended.  The write up has been edited as advised. The revised manuscript now appears as “*This work was funded by the Swedish Research Council (external to my organization), (Grant 2019-04256), Makerere University (internal) and the MakRif Project (internal). There was no other additional external funding received for this study. The content is solely the responsibility of the authors and does not necessarily represent the official views of Swedish Research Council and Makerere University or the MakRif Project. The funders provided support in the form of tuition waiver and research expenses, but did not have any additional role in the study design, data collection and analysis, decision to publish, or preparation of the manuscript”.*  Thanks so much for the caution. We have deleted the information on funding in the Acknowledgement statement. The write up now appears as “*The lead author along with the co-authors extend their gratitude to the participants, doctoral committee and the research team that enable this study to become a reality. This study was undertaken as part of the lead author’s doctoral dissertation.*  .  The amendment has been made in the write up as advised.  The write up has been edited to avail all the additional information. The write up now appears as *“all data underlying the study’s findings have been availed in the manuscript. Additional supplementary information such as the consent forms, interview guide and administrative clearance forms have been availed as supplementary files”.*  The reference list has been edited and reviewed as recommended.  Thanks so much for your kind remarks.  The word “dare” has been changed to “dire”.  The sentence having “yet” has been improved. In the revised manuscript, the write up now appears as “*Despite proven effectiveness, safety, and failure rate of less than one percent of intrauterine contraception [14], uptake among Ugandan women has stagnated below two percent among married and one percent among sexually active unmarried women[15] for reasons that may not be well documented”.*  The interviews were conducted in either English or Luganda. Clarity has been added to the revised version of the manuscript. The write up now appears as “*Fifteen in-depth interviews were conducted in either English or Luganda, the commonly spoken local language in central Uganda, by two interviewers and two note takers with prior experience in qualitative inquiry*”.  Thanks for the comment. We presented our results as general perceptions of the individual couples in the different in-depth interviews. We should have may be reported the divergent responses in some of the couple interviews on some of the issues. We missed out of some of these aspects to avoid lengthening the write up. We have however acknowledged it as a limitation of our study.  Inability to interview the women and their partners separately has also been acknowledged as a limitation.  Thanks so much for the kind remark.  The sentence has been revised as recommended by the reviewer. The write up in the revised manuscript now appears as “*Unintended pregnancies, which are either unplanned or mistimed pregnancies [1], continue to be prevalent globally yet with dire economic, health and social consequences [2].”*  Thanks for the recommendation. I have read the recommended articles. The overall utilization of IUDs as per the Uganda Demographic Health Survey 2022, is 2% among married women and 1% among unmarried women. (<https://www.ubos.org/wp-content/uploads/publications/UDHS-2022-Report.pdf>) page 99, **figure 7.1** contraceptive use. This the most recent official communication from the Ministry of Health of Uganda.  Noor reports the overall uptake all methods of immediate postpartum contraception at 15.4%. This might not be the true reflection of IUDs generally. The second paper is about the unmet need for contraception in Uganda of 28.4%. I think the message is different.  The sentence has however been edited. The write up now appears as “*Despite proven effectiveness, safety, and failure rate of less than one percent of intrauterine contraception [14], uptake among Ugandan women has stagnated below two percent among married and one percent among sexually active unmarried women[15] for reasons that may not be well documented.*”  The write up in the subsequent paragraphs shows from literature review in sub-Saharan Africa, what other researchers found as determinants of uptake of contraception. The existing literature used covers all family planning methods and not necessary postabortion intrauterine contraception. Even in the Ugandan context, the knowledge gaps on IUDs are general and not specific to our study’s context where the IUDs are availed freely to the women undertaking the medical management of first trimester abortions. The write up is an inquiry on whether these prior barriers and facilitators to uptake of contraception generally, could influence the uptake of postabortion intrauterine contraception in central Uganda.  Clarity has otherwise been added to the revised manuscript. The write up now appears as *“Prior women’s experiences on IUD use generally in Uganda[32], showed that though women knew about IUDs, less than 40% of them had the correct information. More than half of the women were not aware of the availability of the method in the neighboring health facility. Nearly 60% of the women believed that IUDs reduce sexual pleasure, damage their wombs and cause cancer [32]. With most of the prior studies interviewing women and men separately on uptake of contraception[17-20], there is uncertainty on whether exploring the women and their partners' perceptions jointly would generate context-based information that would enhance the uptake and utilization of postabortion IUDs. With this contextual background, this study set out to explore women’s and their partners’ perceptions on postabortion intrauterine contraception in central Uganda with the aim of understanding the barriers and motivators to the uptake and use of IUDs.*  Thank you so much for the recommendation. The reference list has been revised as recommended by the reviewer.  Maximal variation means we strategically sampled participants of various backgrounds so as to capture a wide range of perspectives, experiences and phenomenon on the key determinants to the uptake of postabortion intrauterine contraception.  In this study we purposely sampled women and their partners from rural, semi-urban and urban areas. We conducted interviews in either English or Luganda. In so doing, we intentionally interviewed couples of low and high education and socio-economic status. We also sampled couples of varying age brackets, parity and religious backgrounds as indicated in Table 1. The rationale for this was generate as much information as possible from different contextual backgrounds.  The descriptive details of the health facilities are meant to show the readership the different contextual settings (rural, peri-urban and urban) from which the women and their partners were selected. It’s also the journal’s requirement to have these details included in the manuscript for other researchers to replicate the study whenever needed.  To be eligible, the women and their partners had to have opted to use postabortion IUDs, and had to be physically accessible for the in-depth interviews. Women who were unwilling to have their partners involved, were excluded.  As indicated in the manuscript, after conducting 12 interviews, there were no new themes, concepts or insights that were arising from the interviews. We later on conducted three additional interviews and there was redundancy in findings. Since there was theoretical saturation, with no further modification or expansion of the theory, we stopped further data collection. It’s to this end that we conducted the 15 in-depth interviews.  The write up has been improved to offer further clarity. The write up in the revised manuscript now appears as “*Data collection and analysis were conducted concurrently to identify any emerging themes or concepts. The research team ensured that the data collected was thorough and comprehensive. After twelve interviews, three more interviews were conducted and there were no more emerging themes, concepts or insights that were arising from the data. There was redundancy in findings from the data collected. Since there was theoretical saturation, with no further modification or expansion of the theory, we assumed data saturation and stopped the interviews.”*  *We used lead research nurses at the five health facilities to identify potential participants who were invited to participate in the study. Clarity has been added in the revised manuscript to offer details of the recruitment process. The write up now appears as “Lead research nurses at the five health facilities helped to purposively sample women and their partners in the RCT based on the women’s and their partners’ knowledge and experience with postabortion IUDs. We aimed at interviewing women and their partners at various points within the six months of utilizing the IUDs. We also purposively sampled women and their partners of different educational backgrounds and residence from urban, semi-urban and rural areas. The women’s and their partners’ socio-demographic details such as phone contacts, places of residence and next of kin, were retrieved from the databases. Phone calls or text messages were used to invite potential participants for the interviews. Information about the study was shared in a translated informed consent highlighting the study objective, selection criteria, risks and benefits involved while participating in the study and confidentiality commitments.*  *Once a verbal consent was offered by the potential participants, a time for the interview for the women and their partners was fixed based on the women’s and their partners’ convenience thereafter. After obtaining written consent, we conducted joint women’s and their partners’ interviews in quiet rooms free from interference at the health facilities.”*  We used both English and Luganda to target women and their partners of different education and socio-economic status. Luganda is the most commonly used local language in central Uganda especially by people of low socio-economic and education backgrounds.  In so doing, we could enhance the generalizability of our findings to different contextual settings.  We ensured back-to-back translation of the interview guides and the transcriptions from the data collected to ensure that we had no loss or misinterpretation of information. Another member of the research team validated the translation accuracy of the interview transcripts.  The write up has been improved to add more detail. The write up in the revised manuscript now appears as *“We ensured back-to-back translation (English to Luganda) of the data collection tools to minimize on misinterpretation of the data collected*.”  *“The interviews were audio-recorded, transcribed verbatim and translated into English. The authenticity of the translated interview content was validated by someone not part of the study ‘AS’.”*  As indicated in prior research, women of high education and socio-economic status were more likely to use IUDs than women of low social economic status. (*Belay, A.S., H. Sarma, and G. Yilak. Spatial distribution and determinants of unmet need for family planning among all reproductive‑age women in Uganda: a multi‑level logistic regression modeling approach and spatial analysis. Contraception and Reproductive Medicine, 2024.* ***9****(1): p. 4).* Women in union were more likely to use IUDs than single women. (*Abdel-Tawab N, Huntington D, Hassan EO, Youssef H, Nawar L. Effects of husband involvement on postabortion patients’ recovery and use of contraception in Egypt. Postabortion Care: Lessons from Operations Research. Nueva York: Population Council, 1999: p. 16-37.)*  Young single women in sub-Saharan Africa are thought to have multiple sexual partners, which could heighten the risk of PID with IUD use (*Nalwadda G, Mirembe F, Byamugisha J, Tumwesigye NM, Faxelid E. Young peoples’ interface with providers of contraceptive care: a simulated client study in two Ugandan districts. Contraception and Reproductive Medicine, 2016.* ***1****: p. 1-10*.). Young and unmarried women are commonly encouraged to abstain for fears of becoming infertile following IUD use (*Cooke, A. A Systematic Review of Physician-Patient Interactions and the Effect of Health Care Provider Bias and Knowledge on Adolescent Contraception Counseling in Developing Countries and Comprehensive Review: Contraceptive Use and Impact of Physician Counseling for Adolescent Patients of Method Choices and Side Effects in Developing Countries. 2019*.”  The manuscript has been revised to cater for these aspects in the introduction. The write up now appears as *“Women’s age [32-34], marital status [35-37] socio-economic and education background[38] have also been reported to influence the decisions to utilize intrauterine contraception.”*  Thanks so much for the compliment.  We appreciate the caution. In this aspect, we looked at cultural beliefs to understand the power dynamics of that given culture and whether this aspect had any influence on the utilization of IUDs. As an aspect of qualitative inquiry, some of the aspects probed by the interviewers are informed from prior research. This aspect was used to ensure that data saturation and that no other themes could arise from the interviews. We also used peer debriefing to improve the subsequent interviews. It’s to this end that some of the responses were presented to some of the participants rather than barriers shared by some of the participants.  Thanks so much for the caution. We have deleted this aspect of participant recruitment and put the interview guide under study materials. We have as well done an overall proofreading and editing of the manuscript as suggested by the reviewer.  We have added more details to the conclusion. The write up now appears as, *“Understanding the socio-cultural dynamics of women and their partners, is pivotal in the uptake of postabortion IUDs. Perceived barriers like myths and misconceptions, fear of IUD side effects, spouse refusal, transport restrictions, need to be addressed to improve use of postabortion IUDs by women and their partners. Healthcare providers should provide evidence-based family planning counselling and provision for women and their partners, to demystify individual and community misconceptions on IUD use. Male involvement, the assurance of early return to fertility after IUD discontinuation, use of champion couples utilizing postabortion IUDs, and community sensitization outreaches on postabortion IUDs, are nuggets that can be explored to enhance the uptake of IUDs. Policy makers need to encourage pre- and in-service trainings that ensure that the healthcare providers are skilled and knowledgeable in all contraceptives available, to demystify misconceptions on intrauterine contraception.”*  Thanks so much for this feedback.  Thanks so much for this feedback.  Thanks so much for this feedback. | Page 39, line 962-969  Page 38, line 950-953  Page 39, line 970-973  Page 39-48  Page 3, line 50-51, line 68-70  Page 7, line 161-163  Page 37, line 922-925  Page 36-37, line 916-919  Page 3, line 50-51  Page 3, line 68-70  Page 4-5, line 108-121  Page 7, line 166-169  Page 6, line 142-144  Page 8, line 183-190  Page 7-8, line 164-179  Page 7, line 157-159  Page 8, line 181-183  Page 4, line 102-104  Page 7, line 148-159  Page 37, line 927-939 |

Yours truly,

**Dr Herbert Kayiga**

Senior Lecturer/Corresponding Author,

Department of Obstetrics and Gynaecology

Makerere University College of Health Sciences

+256777855063, [hkayiga@gmail.com](mailto:hkayiga@gmail.com)
